# Response to Letter to the Editor

**DOI:** 10.3109/02813432.2014.892305

**Published:** 2014-03

**Authors:** Holgeir Skjeie, Trygve Skonnord, Arne Fetveit, Mette Brekke

**Affiliations:** Department of General Practice, Institute of Health and Society, University of Oslo, Postboks 1130, Blindern, 0318 Oslo, Norway. E-mail: Holgeir.Skjeie@medisin.uio.no

We appreciate the comments by Oscar Heyerdahl and Nils Lystad, on our study of acupuncture for infantile colic [1].

There are three main points reflected on in the comments. First, the steep reduction in crying time in both groups at the start of the treatment period. Second, whether both groups had a significant sensory stimulation due to the adhesive bandage. And third, why did the two previous studies of acupuncture in infantile colic find significant results [2,3], whilst we did not.

First, infantile colic is a complex and poorly understood ailment. There is a multifactorial understanding of the crying and crying behaviour, and parent–child interaction is one of the factors possibly involved. In our study, the observed reductions in crying time, including the steep initial reduction, were mainly placebo effects (Hawthorne effect) on the parents. It is not surprising that the initial treatment period shows the strongest changes. But the needle effects were small, if present, and not clinically relevant in this study with proper randomization, standardized intervention procedure, and true blinding validation.

Second, the speculation on the sensory stimulation of the adhesive bandage reflects a belief in a superficial sensory point-specific effect that we do not share, and that is not proven in any controlled study. One of the two previous studies on infantile colic [2] also used adhesive bandage as blinding procedure and found statistically significant differences. Adhesive bandage as a blinding procedure is not the same as sham acupuncture, which is concerned with complex activation of limbic and higher cortical anticipation/reward-effects in adults [4].

Third, in the two previous studies differences between groups concerning reduction in crying time were similar in magnitude compared with our study. However, our end point registered the overall difference in crying time during the study period, while the other studies reported on separated specific time periods and on different parts of the day. The reported statistically significant differences in changes in crying time between groups in these two positive studies did not cover the whole study period but certain aspects and periods. There was no blinding validation in the two other studies, and one of them was not truly randomized [2]. Moreover, they did not discuss the clinical relevance of the observed differences, which we, as general practitioners, consider essential.

Lastly, the notion that the points and method used might be inferior in our study is not consistent with current empirical [5] and scientific [6,7] knowledge.
